# Editorial for “Long-Term Health Effects of the 9/11 Disaster” in *International Journal of Environmental Research and Public Health*, 2019

**DOI:** 10.3390/ijerph16183289

**Published:** 2019-09-07

**Authors:** Robert M. Brackbill, Judith M. Graber, William A. (Allen) Robison

**Affiliations:** 1 World Trade Center Health Registry, New York City Department of Health and Mental Hygiene, New York, NY 10013, USA; 2 Epidemiology Concentration Director, Department of Biostatistics and Epidemiology, Rutgers School of Public Health, Piscataway, NJ 08854, USA; 3 Office of Extramural Research, National Institute for Occupational Safety and Health, Atlanta, GA 30329-4027, USA

The call for articles on the long term health effects of the 11 September 2001 terrorist attacks (9/11) has resulted in twenty-three papers that add a significant amount of information to the growing body of research on the effects of the World Trade Center (WTC) disaster almost two decades later. The attacks on 9/11 were a paradigm altering event in US history and have had major repercussions in the political landscape and response to terrorism. The toll of 9/11 includes the continued impact of accumulated health effects among those who were directly exposed to either the air pollution or re-suspended material that resulted from the collapse of the two WTC towers, and physical injuries or psychological trauma. This includes a wide range of physical and mental health disorders that continue to plague thousands of people 18 years later as well as newly identified conditions emerging as a result of prolonged disease latency. This was recently highlighted by the addition of “The Memorial Glade” at the WTC site that acknowledges illnesses and deaths years after the towers collapsed [1].

The articles in this special issue also demonstrate the importance of the medical monitoring of the wide range of populations exposed to unprecedented levels of physical and psychological insult from the 9/11 attacks. As such, the reports in this issue represent research findings from the clinics supported by World Trade Center Health Programs and the epidemiological follow-up by the World Trade Center Health Registry. Although the majority of the articles represent rescue, recovery, and clean-up workers (12), some other non-rescue recovery groups included in the special issue are residents of Chinatown, just 10 blocks from ground zero (Kung et al. 2019 [2]), and other residents of lower Manhattan (Antao et al. 2019 [3]).

Respiratory and lung problems are among the most prevalent and highly persistent physical health problems arising from 9/11 exposure to dust clouds from the collapsing building and the subsequent re-suspension of dust (Aldrich, 2010 [4]). In this issue, there are nine respiratory-related papers that provide new insights into the long term consequences of lung damage from 9/11 exposure not reported in previous research. These papers highlight the chronic and still emerging health sequela of 9/11 exposure. An analysis of cleaning practices by residents in lower Manhattan showed that cleaning with dry methods was associated with more types of respiratory symptoms than other cleaning methods (Antao, 2019 [3]). Other papers delved into the underlying physical and biological aspects of pulmonary illness among persons exposed to 9/11 (Liu, 2019 [5]; Kwon, 2019 [6]; Pradhan, 2019 [7]). Liu et al. (2019) used chest tomography (CT) and reported that firefighters with high intensity exposure on 9/11 had increased risk of bronchial wall thickening, emphysema, and air trapping. They correlated the CT-identified abnormalities with respiratory symptoms. A second paper also evaluated the role of metabolic syndrome biomarkers (MSBs) among firefighters (e.g., elevated systolic blood pressure and insulin resistance) in airway hyperactivity (Kwon, 2019 [6]). They reported that given 9/11 exposure, having three or more MSBs increased airway hyperactivity beyond that associated with 9/11 exposure. Another paper that evaluated the bronchodilator response among community members exposed to 9/11 found that a proportion of small airway problems were irreversible, which was predicted by the bronchodilator response at initial visits (Pradhan, 2019 [7]). Two other papers evaluated the increased risk of asthma control issues and quality of life as a function of indoor allergens (Rojano, 2019 [8]) and air pollution/irritants (Yung, 2019 [9]). In addition, an emerging respiratory condition, pulmonary fibrosis (PF)—a common long term sequelae of occupational dust exposure—was documented in a paper based on data from the World Trade Center Health Registry for which there was evidence of a dose-response relationship with the level of exposure among rescue/recovery and other 9/11 workers and the likelihood of PF (Li, 2019 [10]).

Three other papers reported additional findings on a condition known as sarcoidosis (Cleven, 2019 [11]; Hena, 2019 [12]) and sarcoid-like granulomatous (Sunil, 2019). Sarcoidosis is a rare autoimmune disease that can affect any organ, but among rescue, recovery, clean-up workers, it has been previously reported as granulomatous disease involving the thoracic organs (Izbicki, 2007 [13]; Jordan, 2011 [14]), primarily among firefighters or other rescue, recovery, or clean-up workers arriving early at the WTC site. One paper in this issue describes sarcoidosis among community members who were patients at the WTC Environmental Health Center (Hena, 2019 [12]). Another paper focused on the genetic predisposition for sarcoidosis in a case control study (Cleven, 2019 [11]). Sunil (Sunil, 2019 [15]) reported a detailed pathology review of sarcoid like granulomatous disease (SGD). Out of seven cases, five were definite SGD and had high exposure to 9/11 WTC dust.

In addition to respiratory disease, other long term adverse health outcomes of WTC-related exposure include neurologic conditions and cancer. Papers in this issue focused on these emerging conditions including peripheral neuropathy (Colbeth, 2019 [16]), paresthesia (Thawani, 2019 [17]), and thyroid cancer (van Gerwen, 2019 [18]; Tuminello, 2019 [19], see Gargano, 2018 [20] for a review of non-respiratory physical health conditions). Two studies focused on neuropathic conditions that included peripheral neuropathy among New York City firefighters and emergency medical workers (Colbeth, 2019 [16]) and parenthesia among community survivors who received treatment at one of the WTC Health programs (Thawani, 2019 [17]). Potential exposures for neuropathic conditions on 9/11/2001 and afterward included heavy metals and complex hydrocarbons. Both studies used the self-reporting of unusual and painful sensations such prickling, burning, or aching pain in the limbs. Colbeth et al. reported a 35% increase in the likelihood of peripheral neuropathy symptoms among those with the highest 9/11 exposure versus low/no exposure. Similarly, Thawani et al. reported a significant hazard ratio of 1.4 for parenthesia among persons who had a job that required cleaning-up materials resulting from building fires and buildings collapsing. The physical health outcome of cancer was represented by two papers on thyroid cancer (Tuminello, 2019 [19]; van Gerwen, 2019 [18]). Thyroid cancer has been identified as a cancer with a higher expected incidence among potential WTC-exposed persons (Zeig-Owens, 2011 [21]; Li, 2016 [22]; Solan et al., 2013 [23]). Tuminello (2019) evaluated the possibility that increased surveillance for thyroid cancer among WTC survivors could account for the elevated thyroid cancer incidence. In another study (van Gerwin, 2019 [18]) that evaluated thyroid cancers derived from the same population, the authors compared the pathological characteristics of cancer tumors of WTC exposed to non-WTC cases in order to assess whether there were more false positives among the WTC exposed that would suggest a surveillance bias.

The high prevalence of adverse mental health, especially post-traumatic stress disorder (PTSD), has been documented among survivors of 9/11 (Brackbill, 2009 [24]; Stellman, 2008 [25]), in addition to the persistence of PTSD (Pietrzak, 2014 [26]; Maslow, 2015 [27]; Welch, 2016 [28]). A number of papers in this issue addressed the characteristics of those receiving or not adequately receiving mental health treatment and some measurement of the effectiveness of treatment (Jacobson, 2019 [29]; Kung, 2019 [2]; Rosen, 2019 [30]; Bellehsen, 2019 [31]). Based on data from the World Trade Center Health Registry (WTCHR), 38% of enrollees reported they had utilized mental health counseling or therapy sometime in the 15 years after 9/11, with younger persons more likely to seek counseling, but older persons perceiving treatment to be helpful (Jacobson, 2019). Those with persistent PTSD perceived treatment to be less helpful. Another paper also used WTCHR information to characterize unmet mental health care needs for a specific sub-group of Asian WTCHR enrollees, who typically underutilize mental health services (Kung, 2019 [2]). Among the 2300 Asian WTCHR enrollees included in the study, 12% said that they had an unmet mental health care need, for whom 69% reported attitudinal barriers (e.g., I do not need to see a doctor) to utilizing mental health care, 36% said there were cost barriers (e.g., lack of health insurance), and 28% had access barriers (e.g., where to go for doctor, childcare, transportation issues). Two other 9/11 mental health papers used information on patients enrolled in a community WTC Health Program (Rosen, 2019 [30]) and rescue/recovery worker health program (Bellehsen, 2019 [31]). Among patients who reached the criteria for PTSD at the first visit, 77% continued to meet the criteria for PTSD 3 to 4 years later (Rosen, 2019 [30]). Further analysis indicated that some reduction in PTSD symptoms was associated with treatment. The second paper evaluated the extent to which patients were receiving evidence-based treatment (EBT) by community health providers. Like the Rosen et al. paper, they employed baseline and follow-up information in addition to providers reporting their use of EBT. However, after an independent review, 12% of the patients were likely to have received full EBT, and another 40% received some elements of EBT.

Some papers in this issue fittingly addressed the long-term effects of 9/11 exposure on both physical and mental functioning. For instance, Brackbill et al. (2019) [32] assessed the self-reported physical and mental health functioning of persons who were injured on 9/11 15 years after the attack. The severity of injury was associated with physical functioning, but not with mental health functioning; PTSD history also had a significant additive influence on the effect of injury on physical functioning. Using a more objective measure of functionality referred to as handgrip strength, which is a measure of general health status and biomarker of aging, Mukherjee (2019 [33]) reported that rescue/recovery workers with probable PTSD had significantly lower HGS than those without PTSD or depression. Apart from functionality and physical loss, there is concern that persons exposed to 9/11 could be at greater risk of cognitive impairment, memory loss, or confusion at a faster rate than would be expected normally with age. Seil (2019) [34], using the WTC Health Registry data, derived levels of protective factor or cognitive reserve (based on educational level, employed or not, social support, and level of physical activity) for cognitive impairment and found that higher levels of cognitive reserve were associated with less self-reported memory loss for both persons with and without a history of PTSD. Two other aspects of quality of life are represented by papers on early retirement and post-2019 (Yu, 2019 [35]). Among the Lower Manhattan residents and area workers, a history of PTSD and the number of 9/11 related chronic conditions were associated with early retirement (retired before 60). In addition, income loss among those who retired was more likely among those with the highest level of exposure. In the quality of sleep study, it was reported that 9/11 related co-morbidities including gastroesophageal reflux disease, chronic rhinosinusitis, PTSD, anxiety, and depression were associated with a great proportion of sleep related complaints (Ayappa, 2019 [36]). With the presence of these co-morbidities, apnea had no significant impact on sleep quality.

The papers in this special issue clearly document the continued long term effects of the September 11, 2001 WTC disaster on a wide range of health and quality of life issues. They underscore the need for ongoing health monitoring of these highly exposed populations while also representing the cutting edge research on subject areas from the biological underpinnings of 9/11 related respiratory disease to the effectiveness of treatment for mental health problems related to 9/11. This work continues to inform the World Trade Center Health Program for those most affected by the disaster. While this is a uniquely exposed population, this large body research will inform responses to, and the monitoring of, populations exposed to future human caused and natural disasters.
